# Novel Software-Assisted Hemodynamic Evaluation of Pelvic Flow During Chemoperfusion of Pelvic Arteries for Bladder Cancer: Double- Versus Single-Balloon Technique

**DOI:** 10.1007/s00270-016-1296-3

**Published:** 2016-01-27

**Authors:** Kiyohito Yamamoto, Kazuhiro Yamamoto, Go Nakai, Haruhito Azuma, Yoshifumi Narumi

**Affiliations:** Department of Radiology, Osaka Medical College, 2-7, Daigakumachi, Takatsuki-City, Osaka 569-8686 Japan; Department of Urology, Osaka Medical College, 2-7, Daigakumachi, Takatsuki-City, Osaka 569-8686 Japan

**Keywords:** Invasive bladder cancer, Double balloon-occluded arterial infusion, Syngo iFlow

## Abstract

**Purpose:**

Approximately 83 % of patients with bladder cancer have achieved a complete response after undergoing a novel bladder preservation therapy involving balloon-occluded intra-arterial infusion chemotherapy (BOAI) using a four-lumen double-balloon catheter, known as the Osaka Medical College regimen. This study aimed to show the quantitative difference in hemodynamics of the bladder arteries using syngo iFlow (Siemens Healthcare, Erlangen, Germany), which provides an automatic tool for quantitative blood flow analysis between double BOAI (D-BOAI) and conventional single BOAI (S-BOAI).

**Materials and Methods:**

Fifty patients were included. The catheters were introduced into both posterior trunks of the internal iliac arteries via contralateral femoral artery access. A side hole between the distal and proximal balloons was placed at the origin of each bladder artery to allow clear visualization of angiographic flow of the injected agent into the urinary bladder. Digital subtraction angiography was used during analysis with the syngo iFlow to evaluate the hemodynamics of the contrast medium in the pelvic arteries during BOAI. The comparative change in the amount of contrast medium in the bladder arteries between D-BOAI and S-BOAI was assessed using syngo iFlow.

**Results:**

One-hundred pelvic sides were analyzed. The amount of contrast medium in the bladder arteries using D-BOAI was more than twice that using S-BOAI (right, 3.03-fold; left, 2.81-fold).

**Conclusion:**

The amount of contrast medium in the bladder arteries using D-BOAI was higher than that using conventional S-BOAI. This may increase the anticancer drug concentration in the affected bladder, leading to a good clinical response.

## Introduction

The gold-standard therapy for locally invasive bladder cancer has long been radical cystectomy with pelvic lymph node dissection. However, approximately 50 % of all patients with invasive bladder cancer die, and the outcome of this disease is closely related to its pathologic stage [1]. A highly effective but minimally invasive therapy that conserves the bladder is therefore needed. Combined treatment involving radical transurethral resection, chemotherapy, and radiation therapy has been attempted as an alternative approach for patients who require cystectomy.

A novel bladder preservation therapy known as the Osaka Medical College (OMC) regimen has resulted in good clinical responses [1–9]. This regimen adopts double balloon-occluded arterial infusion (D-BOAI) using an original four-lumen double-balloon (4L-DB) catheter. The proximal balloon is located in the internal iliac artery upstream of the bifurcation of the superior gluteal artery. The distal balloon is located at the origin of the superior gluteal artery to isolate the anterior trunk of the internal iliac artery, which lies upstream of the target bladder arteries (BAs) between the balloons. We considered this balloon position suitable, given the variable origins of BAs. It is thought that D-BOAI using the double balloons of the 4L-DB catheter allows for accumulation of a higher concentration of the anticancer agent at the tumor site without flow to other tissues than conventional single balloon-occluded arterial infusion (S-BOAI) using a single-balloon catheter.

Previous studies have angiographically confirmed more accumulation of contrast medium in the tumor site with D-BOAI than with S-BOAI, which might be related to the good clinical response. However, no clinical studies have objectively evaluated the hemodynamics associated with BOAI for patients with bladder cancer. The syngo iFlow (Siemens Healthcare GmbH, Erlangen, Germany) is a software application that uses all of the scenes in a given digital subtraction angiography (DSA) sequence to generate a color map that displays the time of maximum opacification. The further function of iFlow allows retrieval of time–intensity curves for a given point or region of interest (ROI) on the color map. Strother et al. previously described the underlying mathematic model for iFlow [10]. The syngo iFlow has recently enabled analysis of the difference in hemodynamic statuses of the pelvic vessels between D-BOAI and S-BOAI. Therefore, the present study aimed to show the quantitative difference in the hemodynamics of BAs between D-BOAI and S-BOAI in patients with bladder cancer.

## Materials and Methods

### Patients

We performed D-BOAI in patients with bladder cancer who presented for radical cystectomy from November 2013 to September 2014 but who wished to preserve bladder function. Eligible patients had histologically confirmed carcinoma in situ or stage T1, T2, T3, or T4 muscle-invasive bladder cancer without distant metastasis. Imaging studies, including chest and abdominal computed tomography, abdominal/pelvic magnetic resonance imaging, and bone scintigraphy, were performed before the start of therapy. Based on the DSA series collected for the 89 patients, 50 patients (40 men, 10 women; mean age 65.6 years; range 46–83 years) were successfully postprocessed using quantitative DSA with syngo iFlow. In all, 39 patients were excluded because severely tortuous arteries prevented placement of the catheter in the correct position. The clinical characteristics of the patients are presented in Table 1.

**Table 1:** Patients’ characteristics

Characteristic	Data
Age in years, median (range)	68 (46–83)
Sex	
Male	39 (78)
Female	11 (22)
Clinical stage	
T-stage before TUR-Bt	
Tis	5 (10)
Ta	1 (2)
T1	7 (14)
T2	31 (62)
T3	5 (10)
T4	1 (2)
Tumor histology	
UC	
G1	1 (2)
G2	12 (24)
G3	34 (68)
Unknown	3 (6)

Data are presented as *n* (%) unless otherwise indicated

*TUR-Bt* transurethral resection of the bladder tumor, *Tis* carcinoma in situ, *UC* urothelial carcinoma

### BOAI Procedure and Imaging Protocol

**Fig. 1:**
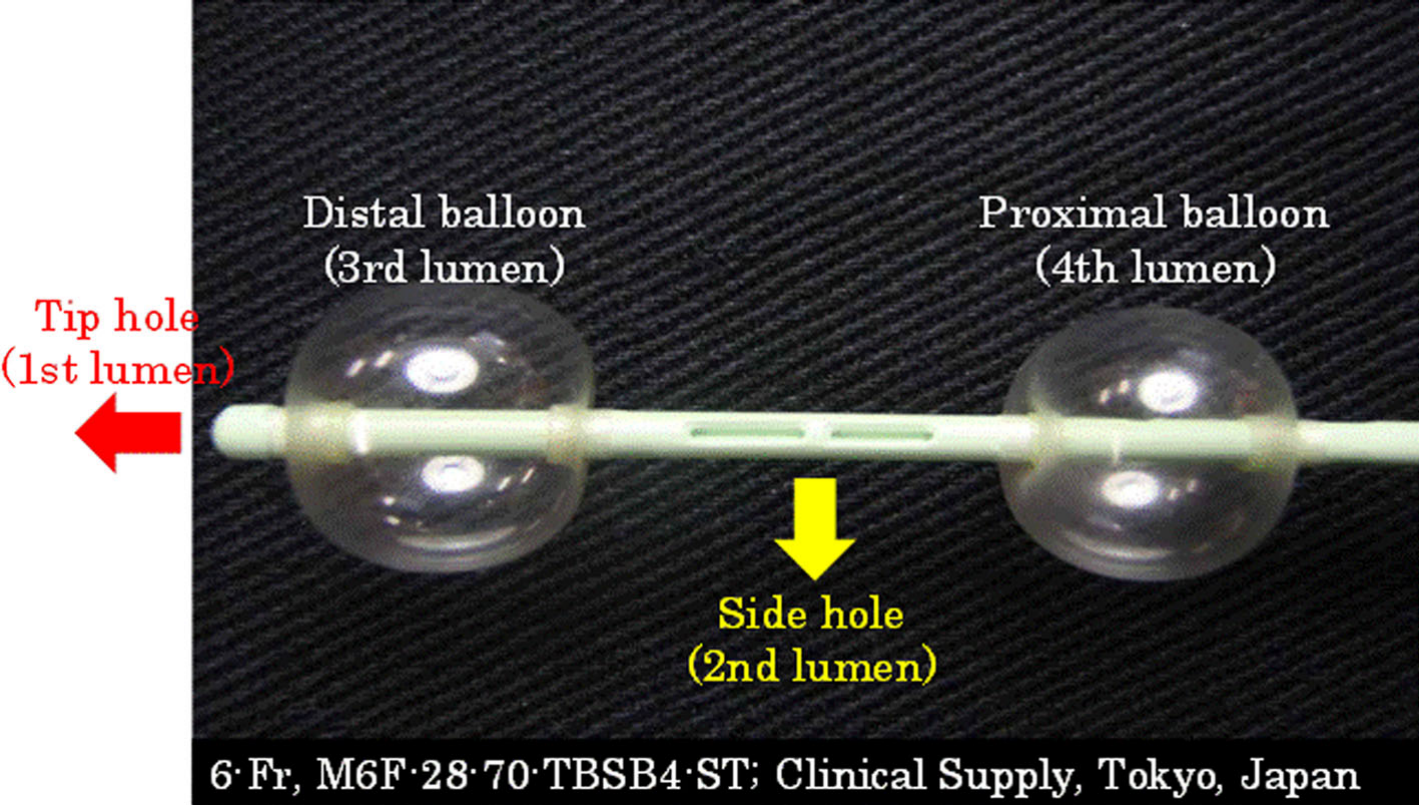
Four-lumen double-balloon catheter. Two of the four lumens are used for balloon inflation. The distance between the two balloons is 4 cm. The diameter of the balloon is 12 mm. An anticancer drug or contrast medium is injected through the side hole

**Fig. 2:**
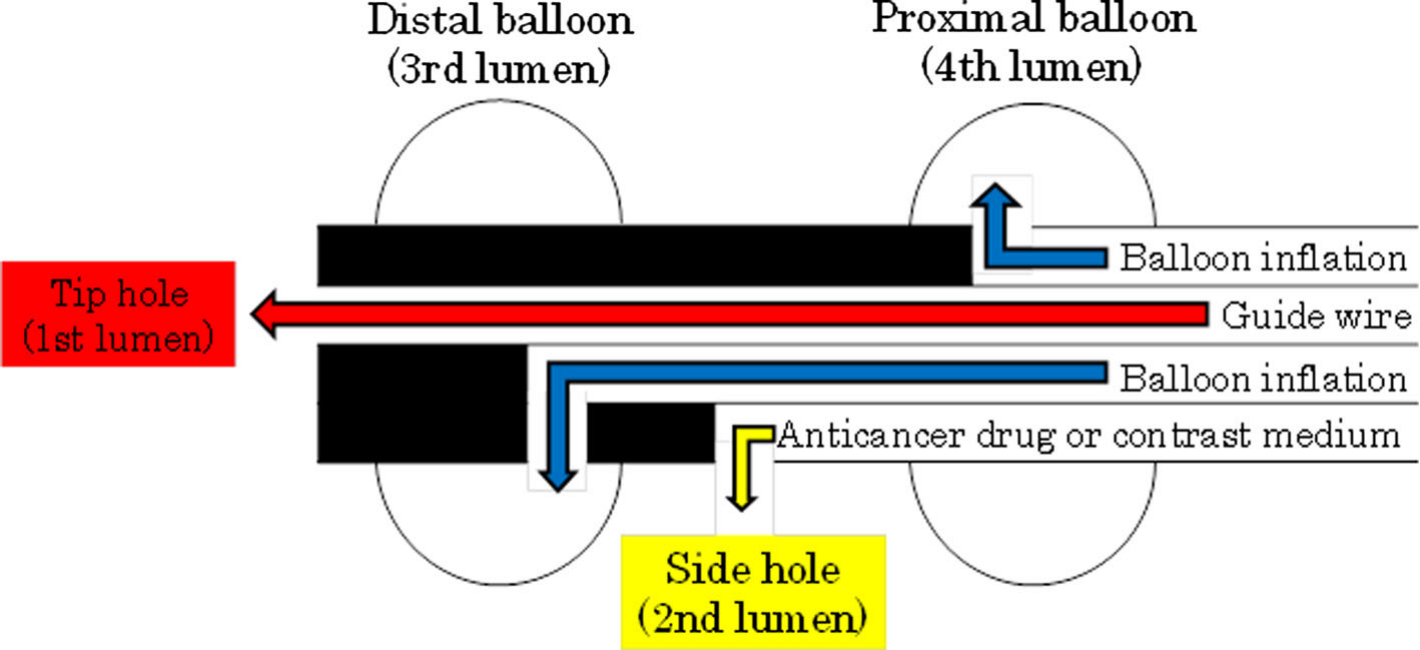
Schema of the four-lumen double-balloon catheter. The first lumen is the tip hole used to insert the guide wire. The second lumen is a side hole used to inject the anticancer drug or contrast medium. The third lumen is used to inflate the distal balloon. The fourth lumen is used to inflate the proximal balloon

For the intra-arterial infusion procedure, we used an intra-arterial catheter equipped with two 6-Fr occlusion balloons (M6F-28-70-TBSB4-ST; Clinical Supply, Tokyo, Japan) (Fig. 1). This catheter, called the 4L-DB catheter, has four lumens. The first lumen is the tip hole used for inserting the guide wire, the second lumen is a side hole used for injection of the anticancer drug or contrast medium, the third lumen is used for inflating the distal balloon, and the fourth lumen is used for inflating the proximal balloon (Fig. 2).

**Fig. 3:**
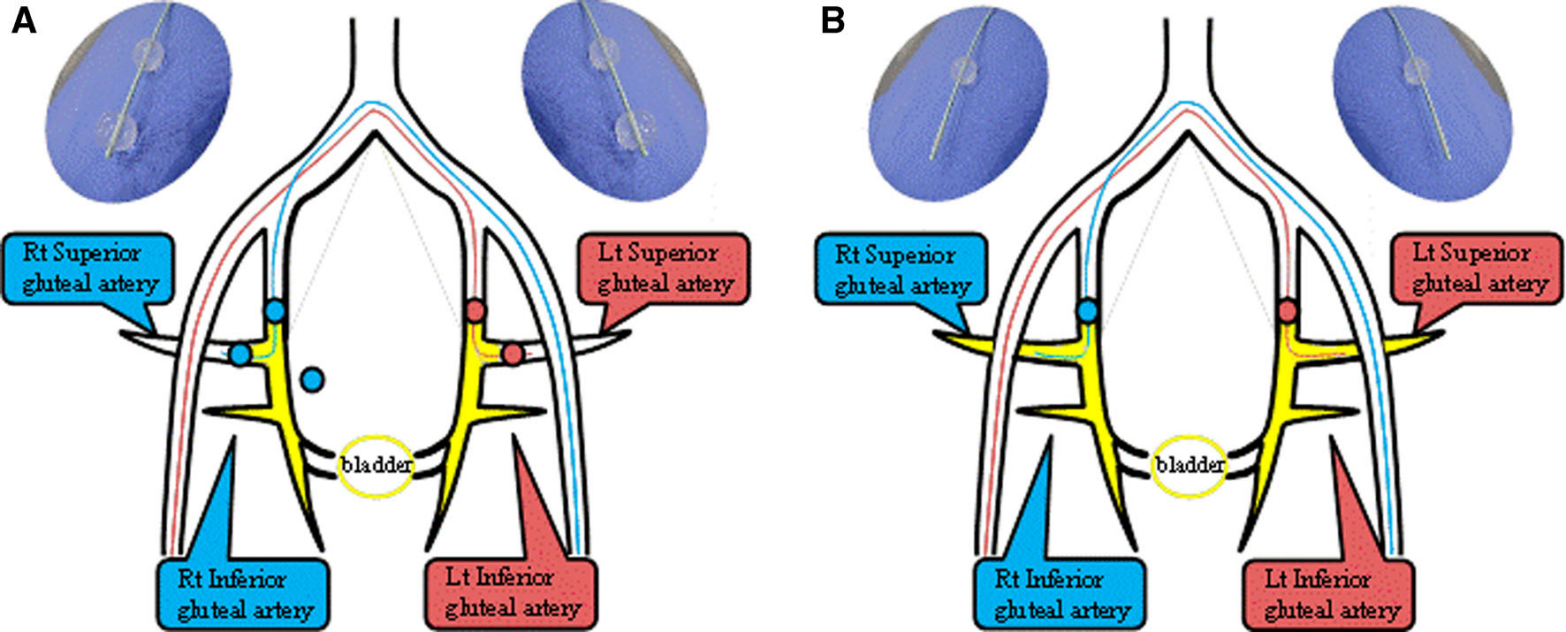
Schema of double and single balloon-occluded arterial infusion (BOAI). **A** Angiography with both distal and proximal balloons inflated [double BOAI (D-BOAI)]. **B** Angiography with only the proximal balloon inflated [single BOAI (S-BOAI)]. Double balloon catheters (6 Fr) are introduced into both superior gluteal arteries via contralateral femoral artery access. Side holes between the distal and proximal balloons are placed at the origin of each bladder artery to allow clear visualization of angiographic flow of the injected agent into the urinary bladder. *Rt* right, *Lt* left

**Fig. 4:**
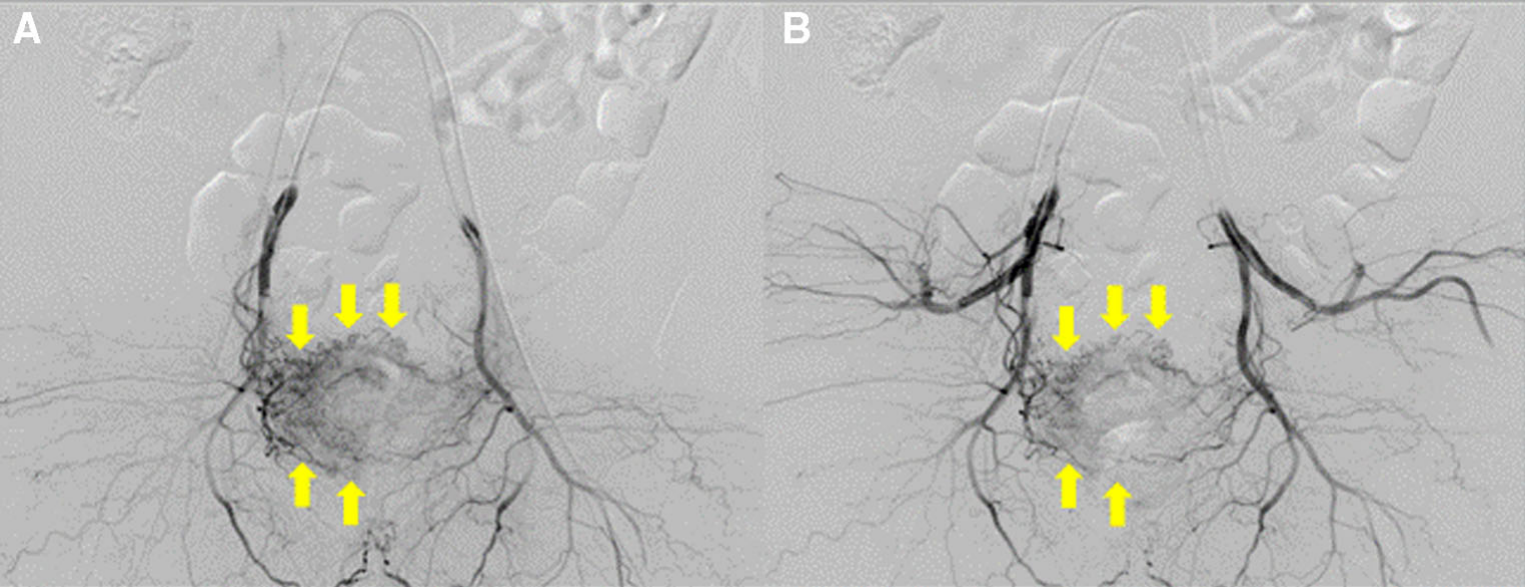
Two patterns of BOAI angiography in a 52-year-old man with invasive urothelial carcinoma. **A** Angiography with both distal and proximal balloons inflated (D-BOAI). **B** Angiography with only the proximal balloon inflated (S-BOAI). Angiography confirms greater accumulation of contrast medium in the bladder arteries with D-BOAI than with S-BOAI (*arrows*)

The catheter was introduced into the posterior trunk of the internal iliac artery via contralateral femoral artery access. After the distal balloon had passed through the furcation of the anterior trunk of the internal iliac artery, the distal and proximal balloons were inflated and immobilized to isolate the anterior trunk of the internal iliac artery, which lies upstream of the target vessels (BAs) between the balloons (Fig. 3). Using DSA, we confirmed that the injected agent did not enter the superior gluteal artery, and there was no back-flow into the internal iliac artery, and the tumor was markedly stained because of active flow of injected contrast medium into the urinary bladder. In the process of intra-arterial infusion chemotherapy as part of the OMC regimen, various amounts of cisplatin (100, 200, or 300 mg) were locally infused through both side holes between the distal and proximal balloons over a 1-h period. We acquired DSA images of D-BOAI and S-BOAI in which only the proximal balloon was inflated (Fig. 4). Although the balloon inflation patterns differed, we used the same 4L-DB catheter for both D-BOAI and S-BOAI.

All angiograms were obtained using the same angiography equipment (Artis zee BA, Siemens AG), and the same injection parameters after bilateral catheters were connected to one high-pressure-resistant extension tube from a contrast medium injector (Mark V ProVis Angiographic Injection System; Medrad, Inc., Warrendale, PA, USA). DSA was performed using the following parameters: 10 mL of iopamidol (370 mgI/mL) injected at a rate of 1.5 mL/s in an anteroposterior position at 4 frames per second. The DSA series were then postprocessed for analysis using the syngo iFlow.

#### Postprocessing of DSA Images

**Fig. 5:**
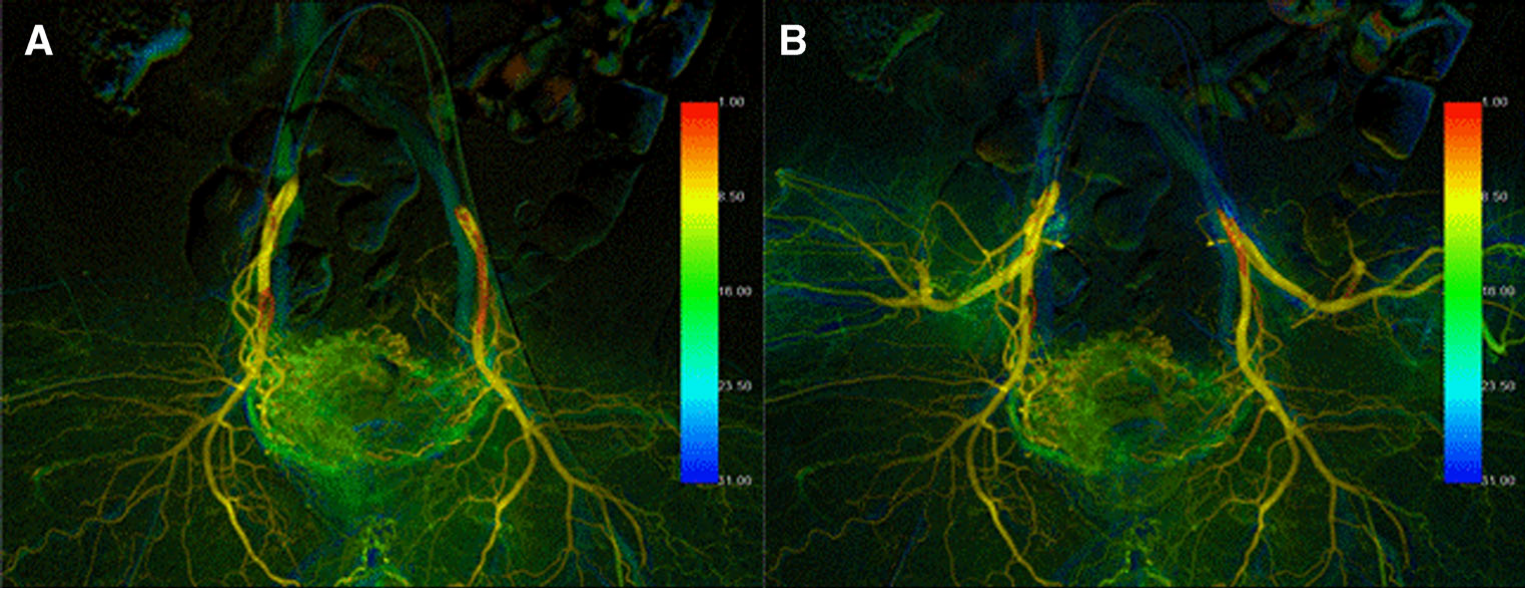
Color-coded images after postprocessing of digital subtraction angiography images of the same patient as shown in Fig. 4 using syngo iFlow. **A** Angiography with both distal and proximal balloons inflated (D-BOAI). **B** Angiography with only the proximal balloon inflated (S-BOAI). The amount of blood flow into the bladder arteries using D-BOAI is quantitatively greater than that using S-BOAI (right, 1.44-fold; left, 1.92-fold)

Both D-BOAI and S-BOAI DSA data (Fig. 4) were transferred to a dedicated image reconstruction workstation (syngo X Workplace; Siemens Healthcare). The hemodynamic parameters were then assessed using syngo iFlow software. The syngo iFlow system provides a single image that shows propagation of the contrast medium through the vessels in color (color-coded single image) by calculating and displaying time–intensity curves for each image pixel (Fig. 5). The time to peak enhancement and the area under the curve (AUC) were identified from each of these pixel-specific curves. The time to peak enhancement is represented by a color (ranging from red to blue) representing early, middle, and late flow in a DSA series. It enables evaluation of the inflow and outflow of contrast medium in dedicated pixels or in ROIs. Hemodynamic parameters of the AUC in the ROI represent the total amount of contrast medium that flows inside the ROI in a DSA series. The color-coded image makes it easy to assess the flow of contrast medium in arteries by showing a complete DSA run in a single image. In this study, we used the AUC value for assessing hemodynamic parameters.

#### Data and Statistical Analysis

The ROIs were placed on both side holes of the catheters, superior gluteal arteries (SGAs), inferior gluteal arteries (IGAs), and BAs on the color-coded single S-BOAI image (Fig. 5). Each ROI placed on the color-coded image of S-BOAI can be automatically transferred to the same position on the color-coded image of D-BOAI from the same patients. The ROI on the side hole of the catheter was defined as the reference ROI (Ref-ROI) when using syngo iFlow. Because bilateral catheters were connected to one tube from the contrast medium injector, each amount of contrast medium delivered through the side hole was regarded as equal. The time–intensity curves of the other ROIs were then analyzed. The AUC was calculated as the sum of the relative density to that of the Ref-ROI at each time point divided by the frame rate. Total relative perfusion was estimated using the calculated AUC from the initial first-pass perfusion to 25 s. The calculated AUC parameter was not an absolute value but a comparative parameter calculated as the AUC of the ROI divided by the AUC of the Ref-ROI. Therefore, the comparative value (CV) for each ROI was calculated as follows:

$$ {\text{CV}} = {\text{AUC of the ROI}}/{\text{AUC of the ipsilateral Ref}} - {\text{ROI}}. $$

Next, we evaluated the change in the CVs for the SGA, IGA, and BA between the D-BOAI and S-BOAI for each side of all 50 patients (total of 100 sides). The change in CV was defined as the ratio of the CVs for the D-BOAI/S-BOAI ROIs. This enabled us to confirm how much more contrast medium ran through the ROI with D-BOAI than with S-BOAI. The calculation for assessment of the change was as follows:

$$ {\text{Change in CV}} = {\text{CV for the ROI with D}} - {\text{BOAI}}/{\text{CV for the ROI with S}} - {\text{BOAI}}. $$

## Results

The results of this study are shown in Table 2. The average changes in the CV for the BA and IGA were 2.92-fold (median 2.01-fold; range 0.62–12.00-fold; right 3.03-fold; left 2.81-fold) and 1.83-fold (median 1.44-fold; range 0.19–10.70-fold; right 1.89-fold; left 1.77-fold), respectively. The average change in the CV for the SGA was 0.41-fold (median 0.31; range 0.09–1.62; right 0.42-fold; left 0.40-fold).

**Table 2:** Changes in CV (CV for the ROI with D-BOAI/CV for the ROI with S-BOAI) for the BA, IGA, and SGA

Change in CV	Average (right) (fold)	Average (left) (fold)	Median (fold)
BA	3.03	2.81	2.01 (range, 0.62–12.00)
IGA	1.89	1.77	1.44 (range, 0.19–10.70)
SGA	0.42	0.40	0.31 (range, 0.09–1.62)

CV (comparative value) = AUC of the ROI/AUC of the ipsilateral reference point

*BA* bladder artery, *IGA* inferior gluteal artery, *SGA* superior gluteal artery, *D-BOAI* double balloon-occluded arterial infusion, *S-BOAI* single balloon-occluded arterial infusion

## Discussion

The syngo iFlow system provides a method for extracting more objective and quantitative information when comparing subjective and qualitative evaluations involving the accumulation of contrast medium during standard DSA. The software tool displays dynamic information in a colorful static image in which different-colored scales mark the history of the contrast medium through vessels. The quantitative measurements of the hemodynamic condition are recorded and depicted using syngo iFlow. Previous reports have also described hemodynamic analysis in the head and neck or liver using syngo iFlow [10–14].

The OMC regimen—a novel therapy for bladder preservation in patients with invasive bladder cancer that involves D-BOAI of an anticancer agent using a 4L-DB catheter—has been used in our hospital for two decades. We have observed improved treatment outcomes using this regimen relative to conventional treatment entailing radical cystectomy with pelvic lymph node dissection [1–9]. In patients with bladder cancer, several feeding arteries are present as branches of the internal iliac artery. Thus, it is difficult to perform selective arterial infusion into these feeding arteries. D-BOAI presumably delivers an extremely high concentration of anticancer agent to the tumor site. Greater accumulation of contrast medium at the tumor site with D-BOAI than with S-BOAI has been confirmed angiographically. However, no reports have described the use of syngo iFlow to assess the quantitative alterations in pelvic vessel hemodynamics as a result of the introduction of D-BOAI versus S-BOAI.

The use of syngo iFlow provides several advantages [14]. First, the application of syngo iFlow does not require additional X-ray exposure because conventional DSA acquisitions can generate color-coded images and obtain quantitative information. Second, syngo iFlow is a real-time tool. Color-coded images with quantitative measurements are obtained immediately after acquisition of the DSA series. Third, hemodynamic conditions and changes can be quantitatively analyzed using parameters such as the AUC through syngo iFlow. This modality focuses not only on alterations in the flow dynamics of the tumor-feeding vessel but also on tumor perfusion, which is represented by the area under the time–intensity curve.

In the present study, it was quantitatively proved that more contrast medium accumulates in the BAs and less accumulates in the SGAs with D-BOAI than with S-BOAI. We assume that the alteration in the accumulation of contrast medium in the BAs with D-BOAI leads to a higher concentration of the anticancer drug in the bladder tissue using the OMC regimen, resulting in a better clinical response.

**Fig. 6:**
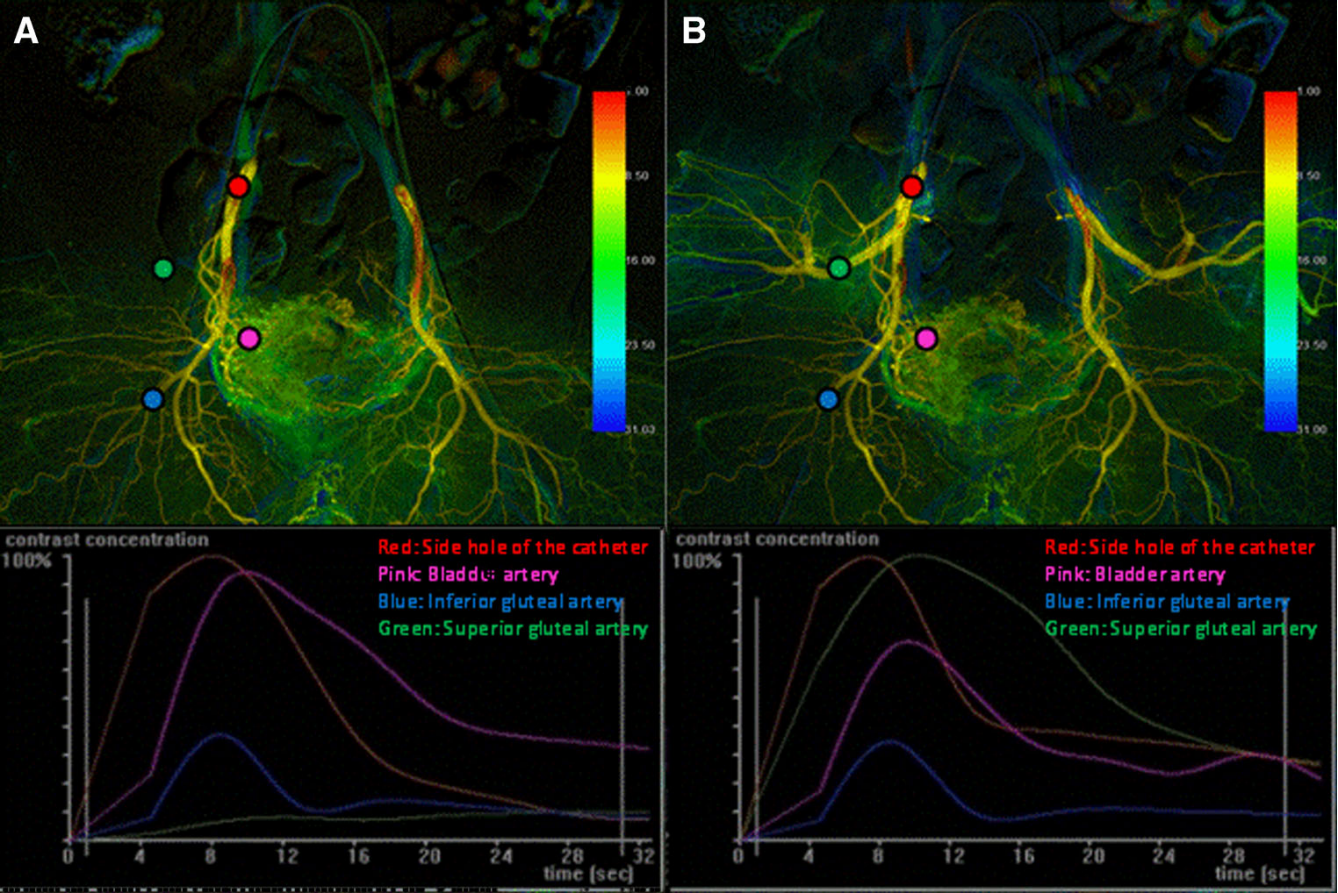
Color-coded images and time–intensity curves after injection of contrast medium. **A** Angiography with both distal and proximal balloons inflated (D-BOAI). **B** Angiography with only the proximal balloon inflated (S-BOAI). *Red, pink, blue,* and *green* regions of interest (ROIs) were placed on the ipsilateral side hole of the catheter, bladder artery, inferior gluteal artery, and superior gluteal artery, respectively. The red ROI (on the side hole of the catheter) was defined as the reference point. The area under the *curve* was calculated as the sum of the relative density to that of the reference point at each time point divided by the frame rate

The present study had several limitations. First, in patients with prominent stenosis of the internal iliac artery or tortuosity of the superior gluteal artery, it may not be possible to introduce a double-balloon catheter into the superior gluteal artery. D-BOAI would not be possible in these cases. Thus, these patients were inevitably excluded from the study. Second, the difference in the clinical response to the treatment with D-BOAI versus S-BOAI could not be evaluated because all patients in this study were treated using D-BOAI. Third, all parameters assessed using syngo iFlow were not absolute values. They were comparative values. Therefore, setting the ROI on the Ref-ROI should be required to calculate the AUC of one particular ROI. In this study, the AUC of the Ref-ROI with D-BOAI was regarded as identical to that with S-BOAI because the Ref-ROI was placed on the side hole of the same catheter. However, as can be seen in Fig. 6, the shape of the time–intensity curve of the Ref-ROI using D-BOAI was different from that using S-BOAI, especially just at the latter part of contrast medium injection. One potential reason is that occlusion of the SGA may cause backward flow in the IGA to compensate for the lack of blood supply to the SGA, leading to the change in blood perfusion of the Ref-ROI as well as the IGA and BA. Furthermore, the change in the AUC of the Ref-ROI affects the other AUCs. Fourth, each ROI does not contain the target vessel fully. This point may lead to inaccuracy of the whole measurement of the perfusion in the target vessel.

In conclusion, compared with S-BOAI, D-BOAI quantitatively increased the amount of contrast medium in the BAs by more than a factor of two. This may result in a higher concentration of anticancer drug in the affected bladder, leading to a good clinical response. However, the long-term clinical outcomes of the patients in this study have not yet been assessed. Further studies are needed to clarify the relation between drug concentration and clinical response.
